# Safety and tolerability of sitagliptin in patients with type 2 diabetes: a pooled analysis

**DOI:** 10.1186/1472-6823-8-14

**Published:** 2008-10-27

**Authors:** Debora Williams-Herman, Elizabeth Round, Arlene S Swern, Bret Musser, Michael J Davies, Peter P Stein, Keith D Kaufman, John M Amatruda

**Affiliations:** 1 Merck Research Laboratories, Rahway, NJ, USA

## Abstract

**Background:**

Sitagliptin, a highly selective dipeptidyl peptidase-4 inhibitor, is the first in a new class of oral antihyperglycemic agents (AHAs) for the treatment of patients with type 2 diabetes. Type 2 diabetes is a life-long disease requiring chronic treatment and management. Therefore, robust assessment of the long-term safety and tolerability of newer therapeutic agents is of importance. The purpose of this analysis was to assess the safety and tolerability of sitagliptin by pooling 12 large, double-blind, Phase IIb and III studies up to 2 years in duration. Methods: This analysis included 6139 patients with type 2 diabetes receiving either sitagliptin 100 mg/day (N = 3415) or a comparator agent (placebo or an active comparator) (N = 2724; non-exposed group). The 12 studies from which this pooled population was drawn represent the double-blind, randomized, Phase IIB and III studies that included patients treated with the clinical dose of sitagliptin (100 mg/day) for at least 18 weeks up to 2 years and that were available in a single safety database as of November 2007. These 12 studies assessed sitagliptin as monotherapy, initial combination therapy with metformin, or add-on combination therapy with other oral AHAs (metformin, pioglitazone, sulfonylurea, sulfonylurea + metformin, or metformin + rosiglitazone). Patients in the non-exposed group were taking placebo, pioglitazone, metformin, sulfonylurea, sulfonylurea + metformin, or metformin + rosiglitazone. This safety analysis used patient-level data from each study to evaluate clinical and laboratory adverse experiences.

**Results:**

For clinical adverse experiences, the incidence rates of adverse experiences overall, serious adverse experiences, and discontinuations due to adverse experiences were similar in the sitagliptin and non-exposed groups. The incidence rates of specific adverse experiences were also generally similar in the two groups, with the exception of an increased incidence rate of hypoglycemia observed in the non-exposed group. The incidence rates of drug-related adverse experiences overall and discontinuations due to drug-related adverse experiences were higher in the non-exposed group, primarily due to the increased incidence rate of hypoglycemia in this group. For cardiac- and ischemia-related adverse experiences (including serious events), there were no meaningful between-group differences. No meaningful differences between groups in laboratory adverse experiences, either summary measures or specific adverse experiences, were observed.

**Conclusion:**

In patients with type 2 diabetes, sitagliptin 100 mg/day was well tolerated in clinical trials up to 2 years in duration.

## Background

Sitagliptin is an oral, once-daily and highly selective dipeptidyl peptidase-4 (DPP-4) inhibitor for the treatment of patients with type 2 diabetes [1]. Inhibition of DPP-4 activity by sitagliptin enhances fasting and postprandial levels of the intact incretins, glucagon-like peptide-1 (GLP-1) and glucose-dependent insulinotropic polypeptide (GIP) [2]. These incretins play a role in glucose homeostasis by increasing insulin release in response to a meal; GLP-1 also decreases glucagon release. Both of these effects are glucose-dependent [3]. In placebo-controlled trials up to 30 weeks in duration, sitagliptin improved fasting and postprandial glycemic control. Sitagliptin, as monotherapy or as add-on therapy to other oral antihyperglycemic agents (AHAs), including metformin, a thiazolidinedione, and a sulfonylurea, has a tolerability profile generally similar to placebo [4-13], although an increase in adverse experiences primarily due to an increase in hypoglycemia was observed when sitagliptin was added to ongoing therapy with a sulfonylurea [10]. Further, in a 52-week, active comparator-controlled trial, the addition of sitagliptin to metformin demonstrated similar efficacy, with a lower incidence rate of hypoglycemia and weight loss versus weight gain, relative to the addition of glipizide to metformin [14].

As type 2 diabetes is a chronic, progressive disease that requires long-term treatment and management, the long-term safety and tolerability of sitagliptin at the usual registered dose of 100 mg/day is of importance. In this report, the safety and tolerability of sitagliptin 100 mg/day were examined in a pooled analysis of patient-level data from 12 double-blind, randomized, Phase IIb and III studies up to 2 years in duration in patients with type 2 diabetes. This pooled analysis provides for a comparison of the safety and tolerability profile of patients treated with sitagliptin 100 mg/day relative to the safety and tolerability profile of patients who participated in these studies but were treated with other therapies (placebo or other oral AHAs), with both groups receiving diet and exercise counseling throughout the studies. The safety and tolerability of dose-adjusted sitagliptin as assessed over 54 weeks in a special populations study of 91 patients with moderate to severe and end-stage renal insufficiency have been reported elsewhere [15].

## Methods

The present analysis focused on the safety and tolerability of the clinical dose of sitagliptin (100 mg/day) approved for use in this broad population of patients with type 2 diabetes [16]. The 12 studies from which this pooled population was drawn represent the double-blind, randomized, Phase IIB and III studies that included patients treated with the clinical dose of sitagliptin (100 mg/day) for at least 18 weeks up to 2 years and that were available in a single safety database as of November 2007. In these studies, patients received sitagliptin as monotherapy or in combination with other oral AHAs, depending upon the particular trial. Patients not receiving sitagliptin received a range of treatments, including placebo, pioglitazone, metformin, sulfonylurea, sulfonylurea + metformin, or metformin + rosiglitazone. This comparison group, patients not receiving sitagliptin, is referred to as the "non-exposed" group. The pooling was conducted by including, from each contributing study, parallel treatment groups of patients with exposure to sitagliptin 100 mg/day (100 mg q.d. [once daily] or 50 mg b.i.d. [twice daily]) that was concurrent to exposure to other treatments (either placebo or active-comparator, Table 1) for generally similar periods of time. Based upon data from a Phase IIb study showing a similar safety and efficacy profile for sitagliptin 100 mg/day administered as either 100 mg q.d. or 50 mg b.i.d. [8], data from these two treatment groups were pooled for this analysis. There were 3 studies in which sitagliptin was administered as 50 mg b.i.d., either alone or in combination with metformin: the 2 Phase IIb studies [6,8], and the study of initial combination therapy with sitagliptin and metformin that simulated the b.i.d. administration of a fixed-dose combination of sitagliptin and metformin [12].

**Table 1:** Studies and treatment arms included in this pooled analysis

Study and Reference*	Study Design	Sitagliptin 100 mg/day Group (N = 3415)	n	Non-exposed Group (N = 2724)	n
**P010: **twice-daily dose-range finding [6]	106-week active-controlled period	• Sitagliptin 50 mg b.i.d. switched to 100 mg q.d.	122	• Glipizide	123

**P014**: once-daily dose-range finding [8]	12-week placebo-controlled period and 94-week active-controlled period	• Sitagliptin 100 mg q.d.	110	• Placebo (12 weeks) switched to metformin (94 weeks)	111
		• Sitagliptin 50 mg b.i.d. switched to Sitagliptin 100 mg q.d.	111		

**P019: **placebo-controlled add-on to pioglitazone study [11]	24-week placebo-controlled period	• Sitagliptin 100 mg q.d. + pioglitazone	175	• Placebo + pioglitazone	178

**P020: **placebo-controlled add-on to metformin study [9]	24-week placebo-controlled period and 80-week active-controlled period	• Sitagliptin 100 mg q.d. + metformin	464	• Placebo + metformin (24 weeks) switched to glipizide + metformin (80 weeks)	237

**P021**: placebo-controlled monotherapy study [4]	24-week placebo-controlled period	• Sitagliptin 100 mg q.d.	238	• Placebo	253

**P023: **placebo-controlled	18-week placebo-controlled period and 36-week active-controlled period	• Sitagliptin 100 mg q.d.	205	• Placebo (18 weeks) switched to pioglitazone (36 weeks)	110

**P024: **active-comparator controlled add-on to metformin study [14]	104-week active-controlled period	• Sitagliptin 100 mg q.d. + metformin	588	• Glipizide + metformin	584

**P035: **placebo-controlled add-on to glimepiride, alone or in combination with metformin [10]	24-week placebo-controlled period and 30-week active-controlled period	• Sitagliptin 100 mg q.d. + glimepiride (± metformin)	222	• Placebo + glimepiride (± metformin) (24 weeks) switched to pioglitazone + glimepiride (± metformin) (30 weeks)	219

**P036: **placebo- and active-controlled study of initial combination use of metformin and sitagliptin[12]	24 week placebo-controlled period and 30-week active-controlled period	• Sitagliptin 100 mg q.d.	179	• Placebo (24 weeks) switched to metformin 1000 mg b.i.d. (30 weeks)	176
		• Sitagliptin 50 mg b.i.d./metformin 500 mg b.i.d.	190	• Metformin 500 mg b.i.d.	182
		• Sitagliptin 50 mg b.i.d./metformin 1000 mg b.i.d.	182	• Metformin 1000 mg b.i.d.	182

**P040: **placebo-controlled monotherapy study [39]	18-week placebo-controlled period	• Sitagliptin 100 mg q.d.	352	• Placebo	178

**P052: **placebo-controlled add-on to metformin and rosiglitazone study [40]	18-week placebo-controlled period	• Sitagliptin 100 mg q.d. + metformin and rosiglitazone	181	• Placebo + metformin and rosiglitazone	97

**P053: **placebo-controlled add-on to metformin study [13]	30-week placebo-controlled period	• Sitagliptin 100 mg q.d. + metformin	96	• Placebo + metformin	94

*References are for the initial phases of the studies that had extension or continuations phases. Studies P019, P040, and P053 did not have continuation phases. For Studies P052, patients continued their sitagliptin or placebo treatment in the currently ongoing continuation phase.

This safety analysis used patient-level data from each study for the evaluation of clinical and laboratory adverse experiences. Adverse experiences were encoded in these studies using the MedDRA (Medical Dictionary for Regulatory Activities; version 10.0) system, a medically validated terminology database developed by the International Conference on Harmonisation. Within this dictionary, adverse event terms are grouped by type of adverse experience (e.g., "Infections and Infestations" or "Investigations") or by body system (e.g., "Gastrointestinal [GI] Disorders" or "Cardiac Disorders"), with groupings referred to as "System Organ Classes" or SOCs. For adverse experiences reported by SOC, a patient was counted once if he/she experienced any adverse experiences included within the SOC (e.g., a patient with the separately reported adverse experiences of 'nausea' and 'abdominal pain' is included separately in the lines for each specific adverse experience but counted only once in the listing for the broader "GI Disorders" SOC, which summarizes the proportions of patients who experienced at least one GI adverse experience). Adverse experiences were expressed as incidence rates (i.e., number of patients with an event divided by total number of patients exposed). Differences in incidence rates between treatment groups and 95% confidence intervals (CI) were calculated using Wilson's score method [17], and clinical adverse experiences with between-treatment group differences in incidence rates for which the 95% CI excluded zero were tabulated. To provide more detail on the overall safety of sitagliptin, specific clinical and laboratory adverse experiences reported for at least 1% of patients in any group were summarized. Since drug-related and serious clinical adverse experiences are generally reported less frequently, these events were summarized using lower cut points (i.e., 0.5% and 0.2% of patients, respectively).

In most studies included in this analysis, glycemic rescue therapy was to be implemented based upon protocol-specified hyperglycemic criteria. The primary analysis in this pooled safety population focused on the results excluding data obtained from time points after a patient initiated rescue therapy to avoid the confounding influence of the rescue therapy.

To assess the robustness of the conclusions from the primary analysis, the results were also analyzed by including data obtained from time points after a patient initiated rescue therapy. An additional analysis, evaluating the number of events per 100 patient-years in study (event rates), was also performed.

### Potential Mechanism-Based Adverse Experiences

Delayed gastric emptying associated with pharmacologic levels of intact GLP-1 has been associated with increased incidences of GI-related adverse experiences such as nausea and vomiting [3]. Since sitagliptin increases intact GLP-1 and GIP levels by 2- to 3-fold [2,18], certain GI adverse experiences were pre-specified for statistical analysis (including diarrhea, nausea, vomiting and abdominal pain [including upper and lower abdominal pain and abdominal/stomach discomfort]) in the sitagliptin development program. Further, since cases of pancreatitis have been reported in association with treatment with a GLP-1 analogue [19,20], this GI adverse experience was also of interest in this pooled analysis.

The DPP-4 enzyme (also referred to as the protein CD26) is expressed on activated T cells, and, although the role of DPP-4 enzyme activity in T cell function is not clear, it is unlikely related to the co-stimulatory function of CD26 [21,22]. Animals genetically deficient in CD26 are healthy and fertile without an evident increase in infections [23], and immune function as well as lymph nodes, spleen, and bone marrow in animals treated with a selective DPP-4 inhibitor appear normal [24,25]. Further, in vitro proliferation of human peripheral blood monocytes is unaffected by selective DPP-4 inhibition [24]. Given the possibility of immune alteration with DPP-4 inhibition, however, adverse experiences in the "Infection and Infestation" SOC were specifically reviewed in the present report.

Serious hypersensitivity reactions, including Stevens-Johnson syndrome, have been reported with the use of sitagliptin in the postmarketing environment [16]. While the causal relationship between sitagliptin and such adverse experiences voluntarily reported from a population of unknown size cannot be definitively established, skin-related adverse experiences were examined in the present pooled analysis. Skin-related findings are also of interest, as preclinical administration of some DPP-4 inhibitors other than sitagliptin (e.g., vildagliptin and PHX1149) [26,27] had been associated with dose-dependent necrotic skin lesions when administered to monkeys. These preclinical findings have not been observed when sitagliptin was administered to monkeys [28], and an increased incidence of skin findings consistent with the preclinical lesions has not been reported in patients in controlled clinical studies with DPP-4 inhibitors, including studies with sitagliptin, vildagliptin, alogliptin, and saxagliptin.

It has been theorized that DPP-4 inhibition could lead to increased events of angioedema, particularly in patients concomitantly treated with angiotensin-converting enzyme (ACE) inhibitors (another class of agents with peptidase activity) [29]. Therefore, a potential association between ACE inhibitor use and the incidence of angioedema-related events (see Appendix I for list of events considered angioedema-related) was evaluated. For this analysis, angioedema-related events were evaluated for the periods with and without exposure to an ACE inhibitor. Exposure to an ACE inhibitor was defined as the total days of ACE inhibitor use during the double-blind treatment period. Patients contributed to patient-years of exposure to an ACE inhibitor for the actual period of time that they were reported to have been taking an ACE inhibitor and to patient-years of non-exposure for the actual period of time that they were reported not to have been taking an ACE inhibitor. Data were expressed as event rates for both incident angioedema-related events (all first events of angioedema) and total angioedema-related events (all events, including those occurring more than once for the same patient) per 100 patient-years of exposure.

### Cardiovascular-related Adverse Experiences

Patients with type 2 diabetes are at increased risk for cardiovascular morbidity and mortality [30]. Recently, there has been increased focus on treatments for diabetes and cardiovascular risk. Therefore, the between-group differences in incidence rates of cardiac (e.g., all cardiac-related adverse experiences, including ischemic and non-ischemic events) and ischemia-related adverse experiences were evaluated in this pooled analysis. This analysis presents adverse experience terms as reported by the investigator; terms for ischemia-related events reported for these studies are found in Appendix II. No formal adjudication was performed.

## Results

### Patient Characteristics and Exposure

In this pooled analysis, there were 6139 patients overall, with 3415 in the sitagliptin 100-mg group and 2724 in the non-exposed group. At baseline, patients in the total cohort had an average age of 55 years (range: 19 to 87 years), a mean duration of diabetes of 5.5 years, and a mean A1C of 8.2%. Men comprised 55% of this cohort and the racial/ethnic breakdown was 57.4% White, 16.2% Asian, 15.2% Hispanic, 5.6% Black, and 5.6% "Other". There were no meaningful differences between treatment groups in these baseline characteristics or in the frequency or type of other medical conditions or medications used.

The mean exposure to drug and total patient-years in study were slightly greater in the sitagliptin group relative to the non-exposed group: 307.3 dosing days (range = 1 to 792) relative to 293.6 dosing days (1 to 801), respectively. In the sitagliptin group, 1343 patients were treated for at least 1 year, with 356 of these patients treated for 2 years. The corresponding numbers of patients in the non-exposed group were 981 and 290. The total duration of observation in study was 2994 patient-years for the sitagliptin group relative to 2270 patient-years for the non-exposed group. In these studies up to 2 years in duration, the proportions of patients discontinuing treatment overall were 34.9% in the sitagliptin group and 39.5% in the non-exposed group, with the reasons for discontinuations generally similar between groups (Table 2).

**Table 2:** Overall disposition of the 6139 randomized patients in the sitagliptin and non-exposed groups

	Sitagliptin 100 mg	Non-Exposed
RANDOMIZED, N	3415	2724

		

	n (%)	n (%)

DISCONTINUED*	1191 (34.9)	1076 (39.5)

Reason for discontinuation		

Clinical adverse experience	119 (3.5)	113 (4.1)

Laboratory adverse experience	38 (1.1)	22 (0.8)

Lack of efficacy^†^	458 (13.4)	381 (14.0)

Patient discontinued for other	152 (4.5)	176 (6.5)

Patient moved	33 (1.0)	19 (0.7)

Patient withdrew consent	186 (5.4)	203 (7.4)

Protocol specified discontinuation criteria	48 (1.4)	44 (1.6)

Protocol deviation	58 (1.7)	50 (1.8)

Lost to follow-up	97 (2.8)	65 (2.4)

Site terminated	2 (0.1)	3 (0.1)

*To provide a complete accounting of the 6139 randomized patients, this table includes data from patients after they received glycemic rescue therapy, whereas the primary safety analysis focuses on results excluding data after patients received rescue therapy. These numbers include patients discontinued over periods of up to 2 years, including those patients who underwent glycemic rescue during the placebo-controlled phase and were ineligible in some studies to enter into the continuation phase.

^†^Includes patients not meeting the progressively stricter, protocol-specified, glycemic rescue criteria and/or not meeting the investigator's expectations of glycemic improvement.

### Clinical Adverse Experiences in the Sitagliptin and Non-Exposed Treatment Groups

The following sections report the results from the primary safety analysis. The findings from the primary safety analysis (excluding data after glycemic rescue therapy) were generally consistent with those that included data after rescue therapy and with those examining the event rates per 100 patient-years (data not shown).

Summary measures of the incidence rates of adverse experiences overall, serious adverse experiences, and discontinuations due to adverse experiences were similar in the sitagliptin and non-exposed groups (Table 3). Drug-related adverse experiences and discontinuations due to drug-related adverse experiences were higher in non-exposed patients, primarily due to hypoglycemia in sulfonylurea-treated patients. In a few SOCs, there were small differences in the incidence rates of adverse experiences (Table 4). For the "Blood and Lymphatic System Disorders" SOC, a slightly higher incidence rate of the adverse experience of anemia in the sitagliptin group compared to the non-exposed group (0.4% vs. 0.1%, respectively; between-group difference [95% CI] = 0.3% [-0.0, 0.6]) accounted for a majority of the between-group difference. The between-group difference in incidence rate in the "Metabolism and Nutrition Disorders" SOC was primarily due to a higher incidence rate of hypoglycemia in the non-exposed group (10.9%) (primarily seen in sulfonylurea-treated patients) relative to the sitagliptin group (3.4%) (Table 5). For the "Investigations" SOC, 3 adverse experiences (blood glucose increased, blood glucose decreased, and weight increased) accounted for the higher incidence rate in the non-exposed group relative to the sitagliptin group (Table 6).

**Table 3:** Clinical adverse experience summary

	Sitagliptin 100 mg n (%) (N = 3415)	Non-Exposed n (%) (N = 2724)	Difference in Sitagliptin and Non-Exposed % (95% CI)*
With one or more adverse experiences	2150 (63.0)	1711 (62.8)	0.1 (-2.3, 2.6)

With drug-related adverse experiences^†^	440 (12.9)	483 (17.7)	-4.8 (-6.7, -3.0)

With serious adverse experiences	230 (6.7)	184 (6.8)	-0.0 (-1.3, 1.2)

With serious drug-related adverse experiences^†^	8 (0.2)	8 (0.3)	-0.1 (-0.4, 0.2)

Who died	11 (0.3)	16 (0.6)	-0.3 (-0.7, 0.1)

Discontinued due to adverse experiences	106 (3.1)	101 (3.7)	-0.6 (-1.5, 0.3)

Discontinued due to drug-related adverse experiences	30 (0.9)	40 (1.5)	-0.6 (-1.2, -0.1)

Discontinued due to serious adverse experiences	51 (1.5)	47 (1.7)	-0.2 (-0.9, 0.4)

Discontinued due to serious drug-related adverse experiences	4 (0.1)	4 (0.1)	-0.0 (-0.3, 0.2)

CI = confidence interval

*Positive differences indicate that the incidence rate for the sitagliptin group is higher than the incidence rate for the non-exposed group. "0.0" and "-0.0" represent rounding for values that are slightly greater and slightly less than zero, respectively.

^†^Determined by the investigator to be possibly, probably, or definitely drug-related.

**Table 4:** Summary of clinical adverse experiences by system organ class

System Organ Class	Sitagliptin 100 mg n (%) (N = 3415)	Non-Exposed n (%) (N = 2724)	Difference between Sitagliptin and Non-Exposed % (95% CI)*
Blood and Lymphatic System Disorders	33 (1.0)	11 (0.3)	0.6 (0.1, 1.0)

Cardiac Disorders	136 (4.0)	105 (3.9)	0.1 (-0.9, 1.1)

Congenital, Familial, and Genetic Disorders	7 (0.2)	4 (0.1)	0.1 (-0.2, 0.3)

Ear And Labyrinth Disorders	50 (1.5)	53 (1.9)	-0.5 (-1.2, 0.2)

Endocrine Disorders	9 (0.3)	15 (0.6)	-0.3 (-0.7, 0.0)

Eye Disorders	140 (4.1)	112 (4.1)	-0.0 (-1.0, 1.0)

Gastrointestinal Disorders	659 (19.3)	493 (18.1)	1.2 (-0.8, 3.1)

General Disorders And Administration Site Conditions	259 (7.6)	212 (7.8)	-0.2 (-1.6, 1.1)

Hepatobiliary Disorders	44 (1.3)	27 (1.0)	0.3 (-0.3, 0.8)

Immune System Disorders	32 (0.9)	25 (0.9)	0.0 (-0.5, 0.5)

Infections And Infestations	1179 (34.5)	897 (32.9)	1.6 (-0.8, 4.0)

Injury, Poisoning And Procedural Complications	290 (8.5)	222 (8.1)	0.3 (-1.1, 1.7)

Investigations	143 (4.2)	145 (5.3)	-1.1 (-2.2, -0.1)

Metabolism And Nutrition Disorders	219 (6.4)	373 (13.7)	-7.3 (-8.8, -5.8)

Musculoskeletal And Connective Tissue Disorders	576 (16.9)	434 (15.9)	0.9 (-0.9, 2.8)

Neoplasms Benign, Malignant And Unspecified^†^	76 (2.2)	44 (1.6)	0.6 (-0.1, 1.3)

Nervous System Disorders	433 (12.7)	344 (12.6)	0.1 (-1.6, 1.7)

Pregnancy, Puerperium, and Perinatal Conditions	1 (0.0)	2 (0.1)	-0.0 (-0.2, 0.1)

Psychiatric Disorders	143 (4.2)	121 (4.4)	-0.3 (-1.3, 0.8)

Renal And Urinary Disorders	99 (2.9)	74 (2.7)	0.2 (-0.7, 1.0)

Reproductive System And Breast Disorders	90 (2.6)	84 (3.1)	-0.4 (-1.3, 0.4)

Respiratory, Thoracic And Mediastinal Disorders	279 (8.2)	208 (7.6)	0.5 (-0.8, 1.9)

Skin And Subcutaneous Tissue Disorders	248 (7.3)	169 (6.2)	1.1 (-0.2, 2.3)

Social Circumstances	2 (0.1)	1 (0.0)	0.0 (-0.2, 0.2)

Surgical and Medical Procedures	3 (0.1)	1 (0.0)	0.1 (-0.1, 0.2)

Vascular Disorders	181 (5.3)	141 (5.2)	0.1 (-1.0, 1.2)

CI = confidence interval

*Positive differences indicate that the incidence rate for the sitagliptin group is higher than the incidence rate for the non-exposed group. "0.0" and "-0.0" represent rounding for values that are slightly greater and slightly less than zero, respectively.

^†^For malignant tumors n (%): 31 (0.9%) in the sitagliptin group and 26 (1.0%) in the non-exposed group.

**Table 5:** Clinical adverse experiences for which the 95% confidence intervals around the difference in incidence rate exclude 0

Adverse Experience	Sitagliptin 100 mg n (%) (N = 3415)	Non-Exposed n (%) (N = 2724)	Difference in Sitagliptin and Non-Exposed, % (95% CI)*
**Sitagliptin > Non-exposed**			

Atrial fibrillation^†^	18 (0.5)	5 (0.2)	0.3 (0.0, 0.7)

Asthenia	19 (0.6)	6 (0.2)	0.3 (0.0, 0.7)

Chest discomfort	9 (0.3)	1 (0.0)	0.2 (0.0, 0.5)

Tooth abscess^‡^	27 (0.8)	10 (0.4)	0.4 (0.0, 0.8)

Osteoarthritis	58 (1.7)	24 (0.9)	0.8 (0.2, 1.4)

Acne	7 (0.2)	0 (0)	0.2 (0.0, 0.4)

Dermatitis Contact	24 (0.7)	7 (0.3)	0.4 (0.1, 0.8)

**Non-exposed > Sitagliptin**			

Bradycardia	0 (0)	4 (0.1)	-0.1 (-0.4, -0.0)

Goiter	1 (0.0)	6 (0.2)	-0.2 (-0.5, -0.0)

Change in bowel habit	0 (0)	4 (0.1)	-0.1 (-0.4, -0.0)

Blood glucose decreased	13 (0.4)	28 (1.0)	-0.6 (-1.1, -0.2)

Blood glucose increased	42 (1.2)	51 (1.9)	-0.6 (-1.3, -0.0)

Weight increased	12 (0.4)	20 (0.7)	-0.4 (-0.8, -0.0)

Hypoglycemia	117 (3.4)	296 (10.9)	-7.4 (-8.8, -6.1)

Sinus headache	3 (0.1)	12 (0.4)	-0.4 (-0.7, -0.1)

Prostatitis	3 (0.1)	9 (0.3)	-0.2 (-0.5, -0.0)

Balanitis	0 (0)	4 (0.1)	-0.1 (-0.4, -0.0)

Hyperkeratosis	0 (0)	8 (0.3)	-0.3 (-0.6, -0.1)

CI = confidence interval

*Positive differences indicate that the incidence rate for the sitagliptin group is higher than the incidence rate for the non-exposed group. "0.0" and "-0.0" represent rounding for values that are slightly greater and slightly less than zero, respectively.

^†^When atrial fibrillation and atrial flutter were combined, the incidence rates were 0.5% and 0.3% for the sitagliptin and non-exposed groups, respectively (between-group difference [95% CI] = 0.3 [-0.1, 0.6]).

^‡^When tooth abscess and tooth infection were combined, the incidence rates were 1.3% and 0.9% for the sitagliptin and non-exposed groups, respectively (between-group difference [95% CI] = 0.4 [-0.1, 1.0]).

**Table 6:** Clinical adverse experiences occurring at an incidence rate ≥1% in any group

Adverse Experience	Sitagliptin 100 mg n (%) (N = 3415)	Non-Exposed n (%) (N = 2724)	Difference between Sitagliptin and Non-Exposed, % (95% CI)*
Vertigo	24 (0.7)	27 (1.0)	-0.3 (-0.8, 0.2)

Abdominal Pain	39 (1.1)	33 (1.2)	-0.1 (-0.6, 0.5)

Abdominal Pain Upper	59 (1.7)	33 (1.2)	0.5 (-0.1, 1.1)

Constipation	79 (2.3)	47 (1.7)	0.6 (-0.1, 1.3)

Diarrhea	170 (5.0)	144 (5.3)	-0.3 (-1.4, 0.8)

Dyspepsia	70 (2.0)	40 (1.5)	0.6 (-0.1, 1.2)

Gastritis	36 (1.1)	30 (1.1)	-0.0 (-0.6, 0.5)

Nausea	85 (2.5)	70 (2.6)	-0.1 (-0.9, 0.7)

Toothache	35 (1.0)	33 (1.2)	-0.2 (-0.8, 0.3)

Vomiting	51 (1.5)	34 (1.2)	0.2 (-0.4, 0.8)

Fatigue	56 (1.6)	53 (1.9)	-0.3 (-1.0, 0.4)

Peripheral Edema	62 (1.8)	54 (2.0)	-0.2 (-0.9, 0.5)

Bronchitis	135 (4.0)	83 (3.0)	0.9 (-0.0, 1.8)

Cellulitis	28 (0.8)	26 (1.0)	-0.1 (-0.6, 0.3)

Gastroenteritis	68 (2.0)	48 (1.8)	0.2 (-0.5, 0.9)

Gastroenteritis Viral	29 (0.8)	27 (1.0)	-0.1 (-0.7, 0.3)

Influenza	145 (4.2)	127 (4.7)	-0.4 (-1.5, 0.6)

Nasopharyngitis	244 (7.1)	162 (5.9)	1.2 (-0.1, 2.4)

Pharyngitis	52 (1.5)	35 (1.3)	0.2 (-0.4, 0.8)

Sinusitis	80 (2.3)	60 (2.2)	0.1 (-0.6, 0.9)

Upper Respiratory Tract Infection	265 (7.8)	228 (8.4)	-0.6 (-2.0, 0.8)

Urinary Tract Infection	134 (3.9)	100 (3.7)	0.3 (-0.7, 1.2)

Viral Infection	36 (1.1)	21 (0.8)	0.3 (-0.2, 0.8)

Blood Glucose Decreased	13 (0.4)	28 (1.0)	-0.6 (-1.1, -0.2)

Blood Glucose Increased	42 (1.2)	51 (1.9)	-0.6 (-1.3, -0.0)

Hyperglycemia	34 (1.0)	39 (1.4)	-0.4 (-1.0, 0.1)

Hypoglycemia^†^	117 (3.4)	296 (10.9)	-7.4 (-8.8, -6.1)

Arthralgia	113 (3.3)	92 (3.4)	-0.1 (-1.0, 0.8)

Back Pain	142 (4.2)	108 (4.0)	0.2 (-0.8, 1.2)

Muscle Spasms	38 (1.1)	35 (1.3)	-0.2 (-0.8, 0.4)

Musculoskeletal Pain	54 (1.6)	40 (1.5)	0.1 (-0.5, 0.7)

Myalgia	38 (1.1)	29 (1.1)	0.0 (-0.5, 0.6)

Neck Pain	23 (0.7)	26 (1.0)	-0.3 (-0.8, 0.2)

Osteoarthritis	58 (1.7)	24 (0.9)	0.8 (0.2, 1.4)

Pain in Extremity	84 (2.5)	53 (1.9)	0.5 (-0.2, 1.2)

Dizziness	86 (2.5)	63 (2.3)	0.2 (-0.6, 1.0)

Headache	169 (4.9)	129 (4.7)	0.2 (-0.9, 1.3)

Hypoesthesia	24 (0.7)	31 (1.1)	-0.4 (-1.0, 0.0)

Anxiety	32 (0.9)	27 (1.0)	-0.1 (-0.6, 0.4)

Depression	40 (1.2)	29 (1.1)	0.1 (-0.4, 0.6)

Insomnia	44 (1.3)	35 (1.3)	0.0 (-0.6, 0.6)

Cough	88 (2.6)	73 (2.7)	-0.1 (-0.9, 0.7)

Pharyngolaryngeal Pain	44 (1.3)	34 (1.2)	0.0 (-0.5, 0.6)

Rash	35 (1.0)	24 (0.9)	0.1 (-0.4, 0.6)

Hypertension	110 (3.2)	89 (3.3)	-0.0 (-1.0, 0.8)

CI = confidence interval; *Positive differences indicate that the incidence rate for the sitagliptin group is higher than the incidence rate for the non-exposed group. "0.0" and "-0.0" represent rounding for values that are slightly greater and slightly less than zero, respectively.

^†^Includes studies in which a sulfonylurea was an active comparator or a background agent.

The most commonly reported adverse experiences in either group were hypoglycemia, upper respiratory tract infection, and nasopharyngitis (Table 6). Of these events, only nasopharyngitis occurred more frequently in the sitagliptin group, although the 95% CI around the between-group difference included 0 (between-group difference [95% CI] = 1.2% [-0.1, 2.4]. For specific clinical adverse experiences reported more frequently in a treatment group and for which the 95% CI around the between-group difference excluded 0, there were 7 specific adverse experiences that were higher in the sitagliptin group and 11 that were higher in the non-exposed group (Table 5). The largest between-group difference (7.4%) was observed for the adverse experience of hypoglycemia, which occurred at a higher incidence rate in the non-exposed group; otherwise the between-group differences in incidence rates were small (< 1%; Table 5). In the development program for sitagliptin, an episode of hypoglycemia was defined as an episode with symptoms of hypoglycemia assessed by the investigator as a clinical adverse experience of hypoglycemia based upon review of the patient's hypoglycemia log. Incidence rates of hypoglycemia were based upon all reports of hypoglycemia; a concurrent fingerstick glucose was not required. The incidence rates of hypoglycemia were driven largely by the use of a sulfonylurea as a comparator agent or as background therapy. Of the 296 patients in the non-exposed group having at least one episode of hypoglycemia, 239 (81%) had an episode while treated with a sulfonylurea. For the patients not treated with a sulfonylurea, the incidence rates for hypoglycemia were 2.6% (n/N: 87/3298) and 2.3% (50/2428) in the sitagliptin and non-exposed groups, respectively. The incidence rates of specific clinical adverse experiences occurring in at least 1% of patients in either group are listed in Table 6.

The incidence rate of drug-related clinical adverse experiences overall was higher in the non-exposed group (17.7%) compared with the sitagliptin group (12.9%), primarily due to reports of hypoglycemia and blood glucose decreased. Drug-related adverse experiences that occurred at an incidence rate of at least 0.5% in either group are listed in Table 7.

**Table 7:** Clinical adverse experiences considered to be related to study drug^† ^that occurred at an incidence rate of ≥0.5% in any group

	Sitagliptin 100 mg n (%) (N = 3415)	Non-Exposed n (%) (N = 2724)	Difference between Sitagliptin and Non-Exposed % (95% CI)*
Abdominal Pain Upper	18 (0.5)	11 (0.4)	0.1 (-0.2, 0.5)

Constipation	25 (0.7)	13 (0.5)	0.3 (-0.2, 0.7)

Diarrhea	42 (1.2)	45 (1.7)	-0.4 (-1.1, 0.2)

Dyspepsia	19 (0.6)	13 (0.5)	0.1 (-0.3, 0.4)

Nausea	33 (1.0)	31 (1.1)	-0.2 (-0.7, 0.3)

Fatigue	19 (0.6)	20 (0.7)	-0.2 (-0.6, 0.2)

Peripheral Edema	11 (0.3)	16 (0.6)	-0.3 (-0.7, 0.1)

Blood Glucose	6 (0.2)	16 (0.6)	-0.4 (-0.8, -0.1)
Decreased			

Hypoglycemia^‡^	87 (2.5)	203 (7.5)	-4.9 (-6.1, -3.8)

Dizziness	17 (0.5)	13 (0.5)	0.0 (-0.4, 0.4)

Headache	37 (1.1)	29 (1.1)	0.0 (-0.5, 0.5)

CI = confidence interval

*Positive differences indicate that the incidence rate for the sitagliptin group is higher than the incidence rate for the non-exposed group. "0.0" and "-0.0" represent rounding for values that are slightly greater and slightly less than zero, respectively.

^†^Determined by the investigator to be possibly, probably, or definitely drug-related.

^‡^Includes studies in which a sulfonylurea was an active comparator or a background agent.

The incidence rate of serious clinical adverse experiences overall was approximately 7% in each treatment group. A review of specific serious clinical adverse experiences irrespective of the relationship to study drug that occurred at an incidence rate of at least 0.2% did not reveal any notable between-group differences (Table 8).

**Table 8:** Serious clinical adverse experiences irrespective of relationship to study drug that occurred at an incidence rate of ≥0.2% in any group

	Sitagliptin 100 mg n (%) (N = 3415)	Non-Exposed n (%) (N = 2724)	Difference between Sitagliptin and Non-Exposed % (95% CI)*
Coronary Artery Disease	5 (0.1)	7 (0.3)	-0.1 (-0.4, 0.1)

Myocardial Infarction	4 (0.1)	5 (0.2)	-0.1(-0.3, 0.1)

Non-cardiac Chest Pain	4 (0.1)	9 (0.3)	-0.2 (-0.5, 0.0)

Cholelithiasis	6 (0.2)	2 (0.1)	0.1 (-0.1, 0.3)

Pneumonia	4 (0.1)	5 (0.2)	-0.1 (-0.3, 0.1)

CI = confidence interval

*Positive differences indicate that the incidence rate for the sitagliptin group is higher than the incidence rate for the non-exposed group. "0.0" and "-0.0" represent rounding for values that are slightly greater and slightly less than zero, respectively.

### Potentially Mechanism-Based Adverse Experiences

The incidence rates of GI adverse experiences did not meaningfully differ between groups (Tables 4, 5, 6). For the pre-specified GI adverse experiences (diarrhea, nausea, vomiting and abdominal pain [including upper and lower abdominal pain and abdominal/stomach discomfort]), the incidence rates were generally similar between groups (Table 6). In the sitagliptin and non-exposed groups, the incidence rates of pancreatitis (0.1% and 0%, respectively), acute pancreatitis (0% and 0.1%, respectively) and chronic pancreatitis (0.1% and 0%, respectively) were low and not meaningfully different between the groups.

The overall incidence rates and intensity of adverse experiences of infection were similar in the two treatment groups, as, in general, were the frequencies and particular types of infections reported (Table 4 and Table 6). Further, the incidence rates of adverse experiences of infection that were reported as serious or that led to discontinuation were similar between groups. In addition, there was no evident increase in the frequency of specific types of infections suggestive of immune suppression (e.g., herpes zoster) between groups. As noted above, the incidence rate of the adverse experience of nasopharyngitis was slightly higher in the sitagliptin group, but in both treatment groups the events were generally mild in intensity and similar in duration, generally occurring as isolated events and after similar time periods following study initiation, and did not recur more frequently in patients in the sitagliptin group relative to those in the non-exposed group.

The overall incidence rate of adverse experiences in the "Skin and Subcutaneous Tissue Disorders" SOC was not meaningfully different between groups (Table 4). Two adverse experiences (acne and contact dermatitis [with approximately half of the events being "poison ivy/oak"]) were reported more frequently in the sitagliptin group compared with the non-exposed group, whereas hyperkeratosis was reported more frequently in the non-exposed group (Table 5). Rash was the only skin-related adverse experience reported by at least 1% of the patients in either group (Table 6), with an incidence rate of 1.0% in the sitagliptin group and 0.9% in the non-exposed group. No adverse experiences of Stevens-Johnson syndrome were reported in patients in the clinical trials that were pooled for this report; one event of erythema multiforme was reported for a patient in the non-exposed group. No adverse experiences consistent with the lesions observed during the preclinical development of some other (non-sitagliptin) DPP-4 inhibitors were reported.

For the analysis of angioedema-related events and the relationship of these events to concurrent treatment with ACE inhibitors, approximately 33% of patients were on an ACE inhibitor at baseline and approximately 36% were on an ACE inhibitor at any time during the studies (for a total of 1,023 and 779 patient-years on an ACE inhibitor for the sitagliptin and non-exposed groups, respectively). Among the 6139 patients included in this pooled analysis, there were 54 incident angioedema-related events, 16 of which occurred in patients concurrently taking an ACE inhibitor. The number of incident angioedema-related events per 100 patient-years while a patient was on an ACE inhibitor was the same (0.9) in both groups. The total numbers of angioedema-related events per 100 patient-years while a patient was on an ACE inhibitor were also similar in the sitagliptin (1.1) and non-exposed (0.9) groups. For patients not on an ACE inhibitor, the numbers of incident angioedema-related events per 100 patient-years were 1.1 in the sitagliptin group and 1.3 in the non-exposed group.

### Cardiovascular-related Adverse Experiences

The incidence rates of overall and serious adverse experiences in the "Cardiac Disorders" SOC were similar between groups (Table 4), with the overall incidence rates of serious adverse experiences in this SOC being 1.2% in the sitagliptin group and 1.5% in the non-exposed group (between-group difference [95% CI] = -0.3% [-1.0, 0.3]). In an analysis in which ischemia-related adverse experiences overall were assessed, the incidence rates were 2.0% in the sitagliptin group and 2.3% in the non-exposed group (between-group difference [95% CI] = -0.2% [-1.0, 0.5]). For the serious ischemia-related adverse experiences, the incidence rates were 1.1% in the sitagliptin group and 1.5% in the non-exposed group (between-group difference [95% CI] = -0.4% [-1.0, 0.2]). There were 3 patients (0.09%) in the sitagliptin group (2 with ischemic stroke and 1 with myocardial infarction) with a fatal ischemic event compared with 7 patients (0.26%) in the non-exposed group (4 with myocardial infarction, 1 with myocardial ischemia, and 2 with sudden cardiac death).

### Laboratory Adverse Experiences in the Sitagliptin and Non-Exposed Treatment Groups

Summary measures of the incidence rates of laboratory adverse experiences overall, serious laboratory adverse experiences, and discontinuations due to laboratory adverse experiences were similar in the two groups (Table 9). There were no meaningful between-group differences in the number of laboratory adverse experiences reported with an incidence rate of at least 1% (Table 10). For hepatic transaminases, the proportions of patients in the sitagliptin and non-exposed groups with their last measurement (obtained either at time of discontinuation or at the final scheduled study visit) of ALT > 3 times the upper limit of normal (3xULN) were 0.7% and 0.8%, respectively; the proportions with last AST measurement > 3xULN were 0.4% and 0.2%, respectively. The number of patients meeting the definition of Hy's Rule (i.e., an ALT and/or AST measurement > 3xULN and a bilirubin > 2xULN) at any time during the study was 2 in the sitagliptin group (with both cases resolving while continuing treatment; 1 patient had suspected concurrent bacteremia and a history of steatohepatitis, and the other had elevated ALT/AST and alkaline phosphatase prior to randomization) and 1 in the non-exposed group (in a patient with concurrent diagnosis of a liver abscess).

**Table 9:** Laboratory adverse experience summary

	Sitagliptin 100 mg n (%) (N = 3375)	Non-Exposed n (%) (N = 2680)	Difference in Sitagliptin and Non-Exposed % (95% CI)*
With one or more adverse experiences	378 (11.2)	293 (10.9)	0.3 (-1.3, 1.8)

With drug-related adverse experiences^†^	102 (3.0)	78 (2.9)	0.1 (-0.8, 1.0)

With serious adverse experiences	3 (0.1)	0 (0)	0.1 (-0.1, 0.3)

With serious drug-related adverse experiences^†^	0 (0)	0 (0)	0.0 (-0.1, 0.1)

Who died	0 (0)	0 (0)	0.0 (-0.1, 0.1)

Discontinued due to adverse experiences	36 (1.1)	18 (0.7)	0.4 (-0.1, 0.9)

Discontinued due to drug-related adverse experiences	16 (0.5)	9 (0.3)	0.1 (-0.2, 0.5)

Discontinued due to serious adverse experiences	0 (0)	0 (0)	0.0 (-0.1, 0.1)

Discontinued due to serious drug-related adverse experiences	0 (0)	0 (0)	0.0 (-0.1, 0.1)

CI = confidence interval

*Positive differences indicate that the incidence rate for the sitagliptin group is higher than the incidence rate for the non-exposed group. "0.0" and "-0.0" represent rounding for values that are slightly greater and slightly less than zero, respectively.

^†^Determined by the investigator to be possibly, probably, or definitely drug-related.

**Table 10:** Laboratory adverse experiences occurring at an incidence rate of ≥1% in any group

Adverse Experience	Sitagliptin 100 mg n/N (%)	Non-Exposed n/N (%)	Difference between Sitagliptin and Non-Exposed, % (95% CI)*
Alanine Aminotransferase Increased	51/3365 (1.5)	37/2672 (1.4)	0.1 (-0.5, 0.7)

Aspartate Aminotransferase Increased	35/3365 (1.0)	26/2672 (1.0)	0.1 (-0.5, 0.6)

Blood Uric Acid Increased	37/3364 (1.1)	22/2672 (0.8)	0.3 (-0.2, 0.8)

Creatine Phosphokinase Increased	22/819 (2.7)	11/619 (1.8)	0.9 (-0.7, 2.5)

Creatinine Clearance Estimation Decreased	31/3243 (1.0)	14/2551 (0.5)	0.4 (-0.1, 0.9)

Fasting Blood Glucose Increased	52/3370 (1.5)	63/2675 (2.4)	-0.8 (-1.6, 0.1)

Low Density Lipoprotein Increased	5/185 (2.7)	2/169 (1.2)	1.5 (-1.9, 5.1)

Protein Urine Present	19/1541 (1.2)	7/1209 (0.6)	0.7 (-0.1, 1.4)

Urine Microalbumin Present	2/228 (0.9)	9/251 (3.6)	-2.7 (-5.9, 0.1)

CI = confidence interval

Data are number of patients with a laboratory adverse experience/number of patients with laboratory measurement expressed as a percentage (n/N [%]).

*Positive differences indicate that the incidence rate for the sitagliptin group is higher than the incidence rate for the non-exposed group. "0.0" and "-0.0" represent rounding for values that are slightly greater and slightly less than zero, respectively.

## Discussion

In this pooled analysis of clinical studies up to 2 years in duration, treatment with sitagliptin 100 mg/day was found to be well tolerated, with generally similar incidence rates of clinical and laboratory adverse experiences in patients treated with sitagliptin relative to those not exposed to sitagliptin. There was a higher incidence rate of drug-related adverse experiences in the non-exposed group, primarily due to the increased incidence rate of hypoglycemia in studies in which a sulfonylurea was used as an active comparator.

In previously reported placebo-controlled trials with sitagliptin as monotherapy, as initial combination therapy with metformin, or as an add-on therapy to agents not associated with hypoglycemia (e.g., metformin, a thiazolidinedione), the incidence rate of hypoglycemia with sitagliptin was low and similar to placebo [4-9,11-13]. This low incidence rate of hypoglycemia observed with sitagliptin is consistent with its glucose-dependent mechanism of action [31]. When sitagliptin was added to ongoing therapy with a sulfonylurea, there was an increase in the incidence rate of hypoglycemia compared to the addition of placebo [10]. This phenomenon has been observed in trials of other AHAs (e.g., metformin, thiazolidinediones, and exenatide), which themselves are not associated with hypoglycemia, when they are added to a sulfonylurea agent [32-34]. In the present pooled analysis, the aggregate incidence rate of hypoglycemia in the sitagliptin group reflects the incidence rates across many studies with various treatment paradigms, including the study in which sitagliptin was added on to a sulfonylurea.

GI side effects are observed with different AHAs, including metformin and α-glucosidase inhibitors [35]. GLP-1 analogues such as exenatide produce levels of GLP-1 activity that are far in excess of physiologic levels and are associated with an increased incidence of nausea and vomiting [3]. In this pooled analysis, the incidence rates of GI adverse experiences, including nausea and vomiting, were similar in the sitagliptin and the non-exposed groups. This was not unexpected because the increased levels of active GLP-1 and GIP observed with the use of DPP-4 inhibitors remain within a physiologic range [3]. Consistent with these findings, when sitagliptin was co-administered with metformin, the GI adverse experience profile of the combination was similar to that of metformin alone [9,12,13].

Inhibition of DPP-4 with a highly selective compound has been shown not to alter measures of immune function in animals *in vivo *and in human immune cells *in vitro *[24,25]. Moreover, mice completely lacking the CD26 molecule are healthy and fertile [23,36]. Consistent with these observations, in the present pooled analysis there were no meaningful differences observed between treatment groups in the incidence rate, severity, and type of infections. Amori *et al*. suggested an increased risk for certain infections (nasopharyngitis and urinary tract infection) with DPP-4 inhibitors (including sitagliptin and vildagliptin) in a recent meta-analysis of efficacy and safety of incretin-based therapies [37]. In the current analysis, only a small numeric increase in the incidence rate of nasopharyngitis in the sitagliptin group was observed, while the incidence rates of urinary tract infections were similar in the two groups. Differences between the current findings and those of Amori *et al*. may be due to several factors, including the larger cohort of patients followed for a longer duration in the present analysis of clinical trials of sitagliptin. Further, the present analysis included all available clinical trial safety data for the specified populations while the Amori meta-analysis could have only included selected adverse experiences meeting different criteria for inclusion in the source manuscripts referenced.

Researchers have suggested that reduced DPP-4 enzyme activity and DPP-4 enzyme deficiency increase ACE inhibitor-induced angioedema in rats and potentially humans [29,38]. These authors hypothesized that the concomitant use of DPP-4 inhibitors and ACE inhibitors could increase the risk of angioedema-related adverse experiences. The clinical experience reported in the present analysis does not support this hypothetical association. Moreover, a higher rate of angioedema-related events in sitagliptin-treated patients, regardless of ACE inhibitor use, was not observed in the present analysis. Hypersensitivity reactions with administration of sitagliptin (including those considered as severe) have been reported based upon postmarketing surveillance. In the current pooled analysis of events reported from controlled clinical trials, hypersensitivity events occurred with similar incidence rates in patients taking sitagliptin relative to the non-exposed group.

Although the studies in this analysis were not cardiovascular outcome trials, but rather trials assessing the overall safety and efficacy of sitagliptin 100 mg/day, there were no meaningful differences between groups in the incidence rates of cardiac-related or ischemia-related adverse experiences.

## Conclusion

In patients with type 2 diabetes, sitagliptin 100 mg/day was well tolerated as monotherapy, as initial combination therapy, and as add-on therapy in double-blind, randomized clinical studies up to 2 years in duration. The safety of sitagliptin continues to be monitored in ongoing clinical studies and through postmarketing surveillance.

## Appendix 1. List of terms* used in the angioedema-related adverse events analysis

Eye disorders system organ class (SOC)

Eye edema, Eye swelling, Eyelid edema

Immune system disorders SOC

Anaphylactic reaction, Angioedema, Drug hypersensitivity, Hypersensitivity

Skin and subcutaneous tissue disorders SOC

Face edema, Periorbital edema, Swelling face, Urticaria, Urticaria generalized

*From the adverse experiences reported in this pooled analysis, these reported terms were identified as angioedema-related.

## Appendix 2. List of terms* used in the ischemia-related adverse events analysis

Vascular disorders SOC

Aortic arteriosclerosis, Aortic stenosis, Arteriosclerosis, Arteriosclerosis obliterans, Extremity necrosis, Iliac artery stenosis, Intermittent claudication, Leriche syndrome, Peripheral arterial occlusive disease, Peripheral ischemia

Nervous system disorders SOC

Carotid artery disease, Carotid artery stenosis, Cerebellar infarction, Cerebral infarction, Cerebrovascular accident, Ischemic stroke, Lacunar infarction, Thalamic infarction, Transient ischemic attack, Vertebrobasilar insufficiency

Cardiac disorders SOC**

Acute coronary syndrome, Acute myocardial infarction, Angina pectoris, Angina unstable, Arteriosclerosis coronary artery, Coronary artery disease, Coronary artery insufficiency, Coronary artery occlusion, Coronary artery stenosis, Ischemic cardiomyopathy, Myocardial infarction, Myocardial ischemia, Silent myocardial infarction, Ventricular tachycardia

General disorders and administration site conditions SOC

Sudden cardiac death

*From the adverse experiences reported in this pooled analysis, these reported terms were identified as ischemia-related.

**One patient in the non-exposed group with sepsis and multi-organ failure with cause of death reported as 'cardio-respiratory arrest' was not included in the ischemia-related analyses.

## Competing interests

All authors are or were formerly (PPS) employed by Merck & Co., Inc., the manufacturer of sitagliptin. All authors own company stock and, except for PPS, have stock options.

## Authors' contributions

DWH, PPS, KDK, and JMA conceived the design for the analysis. DWH, ER, AS, BM, PPS, KDK, and JMA participated in the design of the analysis. AS and BM performed the statistical analysis. All authors were involved in the interpretation of the analysis. All authors were involved in drafting the manuscript or revising it critically for important intellectual content. All authors approved the final manuscript.

## Pre-publication history

The pre-publication history for this paper can be accessed here:


